# Distribution of phylogenetic groups, adhesin genes, biofilm formation, and antimicrobial resistance of uropathogenic *Escherichia coli* isolated from hospitalized patients in Thailand

**DOI:** 10.7717/peerj.10453

**Published:** 2020-12-02

**Authors:** Nipaporn Tewawong, Siriporn Kowaboot, Yaowaluk Pimainog, Naiyana Watanagul, Thanunrat Thongmee, Yong Poovorawan

**Affiliations:** 1 Faculty of Medical Technology, Rangsit University, Muang, Pathumthani, Thailand; 2 Department of Microbiology, Nopparat Rajathanee Hospital, Khannayao, Bangkok, Thailand; 3 Center of Excellence in Clinical Virology, Department of Pediatrics, Faculty of Medicine, Chulalongkorn University, Pathumwan, Bangkok, Thailand

**Keywords:** Uropathogenic *Escherichia coli*, Phylogenetic group, Adhesin genes, Biofilm, Antimicrobial resistance

## Abstract

**Background:**

Urinary tract infections (UTIs) are the most common bacterial infections and are often caused by uropathogenic *Escherichia coli* (UPEC). We investigated the distribution of phylogenetic groups, adhesin genes, antimicrobial resistance, and biofilm formation in *E. coli* isolated from patients with UTIs.

**Methods:**

In the present study, 208 UPEC isolated from Thai patients were classified into phylogenetic groups and adhesin genes were detected using multiplex PCR. Antimicrobial susceptibility testing was performed using agar disk diffusion. The Congo red agar method was used to determine the ability of the UPEC to form biofilm.

**Results:**

The most prevalent UPEC strains in this study belonged to phylogenetic group B2 (58.7%), followed by group C (12.5%), group E (12.0%), and the other groups (16.8%). Among adhesin genes, the prevalence of *fimH* (91.8%) was highest, followed by *pap* (79.3%), *sfa* (12.0%), and *afa* (7.7%). The rates of resistance to fluoroquinolones, trimethoprim-sulfamethoxazole, and amoxicillin-clavulanate were  65%, 54.3%, and 36.5%, respectively. The presence of adhesin genes and antibiotic resistance were more frequent in groups B2 and C compared to the other groups. Of the 129 multidrug-resistant UPEC strains, 54% were biofilm producers. Our findings further indicated that biofilm production was significantly correlated with the *pap* adhesin gene (*p* ≤ 0.05).

**Conclusion:**

These findings provide molecular epidemiologic data, antibiotic resistance profiles, and the potential for biofilm formation among UPEC strains that can inform further development of the appropriate prevention and control strategies for UTIs in this region.

## Introduction

Urinary tract infections (UTIs) are a common bacterial infection, with 150 million UTI cases observed annually worldwide (Stamm & Norrby, 2001). Uropathogenic *Escherichia coli* (UPEC) is the most common causative agent of both uncomplicated and complicated UTIs, accounting for 75% and 65% of cases, respectively (Flores-Mireles et al., 2015). Clermont and colleagues developed a new polymerase chain reaction (PCR)-based method to classify the eight phylogenetic groups of *E. coli*, of which seven are clustered in *E. coli sensu stricto* (A, B1, B2, C, D, E, and F) and one belongs to *Escherichia* Clade 1 (Clermont et al., 2013). Several studies have reported that phylogenetic groups B2 and D are associated with extraintestinal infection, while the other groups are more prevalent among diarrheagenic and commensal bacteria (Picard et al., 1999; Kumar, Nahid & Zahra, 2017; Ahumada-Santos et al., 2020).

Adherence and colonization are the crucial steps in UTI pathogenesis. UPEC generally use various adhesins to recognize uroepithelium cells and mediate colonization (Flores-Mireles et al., 2015). Type 1 fimbriae consist of a major protein, FimA, that is associated with the ancillary proteins FimF, FimG, and the adhesin FimH, all of which are encoded by the *fim* gene cluster (Orndorff & Falkow, 1984). The P fimbriae are encoded by the *pap* gene cluster, which contains 11 genes (*papA* to *papK*) (Fernández & Berenguer, 2000). P fimbriae promote early colonization of the epithelial cells lining the tubules, while type 1 fimbriae appear to play a role in inter-bacterial binding and biofilm formation (Melican et al., 2011). The S fimbriae are expressed by the *sfa* operon, which was reported to be most often found in *E. coli* strains implicated in human meningitis and septicemia (Antao, Wieler & Ewers, 2009). The P-independent, X-binding fimbrial adhesin encoded by the *afa1* operon mediates specific binding to uroepithelial cells and human erythrocyte receptors (Labigne-Roussel & Falkow, 1988). Different studies have investigated the presence of the adhesion-encoding genes *pap* (P fimbriae), *sfa* (S fimbriae), *afa* (afimbrial adhesin), and *fimH* (type 1 fimbriae) across UPEC strains using multiplex PCR (Rahdar et al., 2015; Dadi et al., 2020; Tarchouna et al., 2013; Shetty et al., 2014).

Currently, the empirical treatment of UTIs is an issue of concern due to the increasing rates of antibiotic resistance. The resistance to trimethoprim-sulfamethoxazole (TMP-SMZ), ciprofloxacin, and amoxicillin-clavulanate (AMC) among UPEC isolates is higher in developing countries (ranging from ∼50% to 85%) than in developed countries (ranging from 3% to 40%) (Kot, 2019). Routine standard antimicrobial susceptibility testing must be performed in order to reduce the rates of inappropriate empirical antibiotic therapy of UTIs and thereby decrease the occurrence of multidrug-resistant (MDR) UPEC (Adamus-Białek et al., 2018).

Biofilms are microbial communities that adhere to various surfaces, and the cells within a biofilm are encased in self-produced extracellular polymeric matrix (Hall & Mah, 2017). The ability of UPEC to form biofilms is important, as biofilms increase antimicrobial agent tolerance and facilitate evasion of the urinary tract host defense, contributing to the evolution of MDR strains and the recurrence of UTIs (Mittal, Sharma & Chaudhary, 2015).

A study of virulence genes and antimicrobial susceptibility patterns of UPEC in southern Thailand was previously reported (Themphachanal et al., 2015), but there is no information on the new classification of phylogenetic groups or the biofilm-forming ability of UPEC. Therefore, the aim of the present study was to determine the phylogenetic groups, adhesin gene distribution, antimicrobial resistance profiles, and biofilm formation ability of UPEC isolated from patients with UTIs in central Thailand. We also investigated the possible correlation between adhesin genes and the ability to form biofilm.

## Materials and Methods

### Ethical approval

*E. coli* strains were isolated from patients with UTI then identified and collected at the Nopparat Rajathanee Hospital as part of the routine microbiological laboratory. The study protocol was approved by the Ethics Review Board (ERB) of the Research Institute of Rangsit University (DPE.No.RSUERB2018-002). All the bacterial strains were acquired with permission from the Director of Nopparat Rajathanee Hospital**.**

### Bacterial strains

The 208 non-repetitive *E. coli* strains isolated from urine specimens of UTI patients between February and May 2018 were used from the current study. *E. coli* strains were isolated from pure cultures and identified in the department of microbiological laboratory in the Nopparat Rajathanee Hospital. The bacteria were confirmed as *E. coli* by considering Gram’s staining morphology, colony characteristic on MacConkey agar (Oxoid, UK), and biochemical properties (Bergey & Holt, 1994). The oxidase test, catalase test, sugar fermentation, motility test, indole production, methyl red test, Voges-proskauer reaction, urease production, citrate utilization, and ornithine and lysine decarboxylase test were used as the standard biochemical testing in our laboratory. The only one isolate from each patient was investigated.

### Characterization of phylogenetic groups and adhesin genes

Bacterial DNA was extracted using the optimized boiling method (Dashti et al., 2009). The phylogenetic groups of *E. coli* were characterized using multiplex PCR according to the protocol previously published (Clermont et al., 2013). Table S1 shows the primer sequences and the size of amplicons. In addition, four adhesin genes, *pap*, *sfa*, *afa*, and *fimH*, were detected in all isolates using multiplex PCR (Yamamoto et al., 1995; Le Bouguenec, Archambaud & Labigne, 1992; Struve & Krogfelt, 1999). The details of the primers and sizes of PCR products are listed in Table S2. The PCR reaction volume contained 15 µl of 2X AmpMaster™ HS-Taq (GeneAll^®^, Korea), 10 pmol/µl of each primer, 3 µl of DNA template, and DNase-free H_2_O to a final volume of 30 µl. Amplification was carried out in the Mastercycler^®^ nexus (Eppendorf, Germany) under the following conditions: initial denaturation at 95 °C for 3 min, 45 cycles of 45 s denaturation at 95 °C, 45 s of primer annealing at 55 °C (to characterize the phylogenetic groups) and 54 °C (to amplify the adhesin genes), 60 s of extension at 72 °C, and further extension for 5 min at 72 °C. PCR products were separated on a 2% agarose gel with a 100-bp DNA ladder (Fermentas, US) and visualized on a UV trans-illuminator.

### Antimicrobial susceptibility testing

Antimicrobial susceptibility tests were performed using the agar disk diffusion method according to Clinical and Laboratory Standards Institute guidelines (CLSI, 2018). The antibiotic disks (Oxoid, UK) ampicillin (10 µg), amoxicillin-clavulanate (20/10 µg), piperacillin-tazobactam (100/10 µg), cefoperazone-sulbactam (75/30 µg), cefazolin (30 µg), cefotaxime (30 µg), ceftriaxone (30 µg), ceftazidime (30 µg), imipenem (10 µg), meropenem (10 µg), ertapenem (10 µg), gentamicin (10 µg), amikacin (30 µg), netilmicin (30 µg), ciprofloxacin (5 µg), levofloxacin (5 µg), norfloxacin (10 µg), trimethoprim-sulfamethoxazole (1.25/23.75 µg), and fosfomycin (200 µg) were used. *Escherichia coli* ATCC 25922 was used as a control in all antibiogram tests. Whether a strain was MDR was determined on the basis of acquired non-susceptibility to at least one agent in three or more antimicrobial categories (Magiorakos et al., 2012).

### Detection of biofilm formation

The biofilm production of all *E. coli* strains was determined using the Congo red agar (CRA) method, as previously published (Neupane et al., 2016; Sm, Jayakumar & Aravazhi, 2016; Tajbakhsh et al., 2016). The medium contains brain heart infusion agar (52 gm/L); sucrose (36 gm/L) and Congo red dye (0.8 gm/L). The tested organisms were cultured on CRA and incubated under the aerobic condition at 37 °C for 24 to 48 h. The six color tones of colonies were categorized as follows: very black, black, almost black, which were interpreted as strong, moderate, and weak biofilm producers, respectively, and bordeaux, red, and very red, reported as non-biofilm producers.

### Statistical analysis

Chi-square test was used for comparisons of proportions the demographic characteristics of patients. The correlations between phylogenetic group, the presence of adhesin genes, biofilm production, and antimicrobial resistance were determined by performing Pearson’s chi-square tests. SPSS version 21 software was used for data analysis (IBM SPSS Inc., Armonk, NY, USA). Results were considered statistically significant if the *p*-value was ≤ 0.05.

## Results

Among 1,926 patients with symptoms of UTI, a total of 208 isolates were identified as *E. coli*. The demographic characteristics of patients infected with UPEC are shown in Table 1. Among the patients, 154 (74%) were female and 54 (26%) were male. Patients were stratified into five different age groups, and those over 65 years represented 63.9% of all patients. The highest number of UPEC samples was isolated from catheter urine samples (150, 72.1%). The highest proportion of UPEC isolates came from the internal medicine ward (80, 38.5%), followed by the emergency room (45, 21.6%), intensive care unit (34, 16.3%), and outpatients (22, 10.6%).

**Table 1:** Demographic characteristics of patients infected with uropathogenic *E. coli* (*N* = 208).

**Parameter**		**No. of isolates (%)**	**Chi-square**	**Degree of freedom**	***p*-value**
**Gender**					
	Female	154 (74.0)	48.08^a^	1	<0.0001
	Male	54 (26.0)			
**Age (years)**					
	<14	11 (5.3)	265.02^b^	4	<0.0001
	15–24	6 (2.9)			
	25–44	15 (7.2)			
	45–64	43 (20.7)			
	≥65	133 (63.9)			
**Type of samples**					
	Midstream urine	58 (27.9)	40.69^a^	1	<0.0001
	catheter urine	150 (72.1)			
**Hospital Unit**					
	Out-patient	22 (10.6)	188.23^c^	7	<0.0001
	In-patient				
	Internal medicine	80 (38.5)			
	ER	45 (21.6)			
	ICU	34 (16.3)			
	Surgery	12 (5.8)			
	Pediatrics	8 (3.8)			
	Stroke	5 (2.4)			
	Burn	2 (1.0)			
**MDR stains**					
	MDR	129 (62.0)	12.02^a^	1	0.001
	Non-MDR	79 (38.0)			

**Notes.**

a 0 cells (.0%) have expected frequencies less than 5. The minimum expected cell frequency is 104.0.

b 0 cells (.0%) have expected frequencies less than 5. The minimum expected cell frequency is 41.6.

c 0 cells (.0%) have expected frequencies less than 5. The minimum expected cell frequency is 26.0.

**Figure 1:**
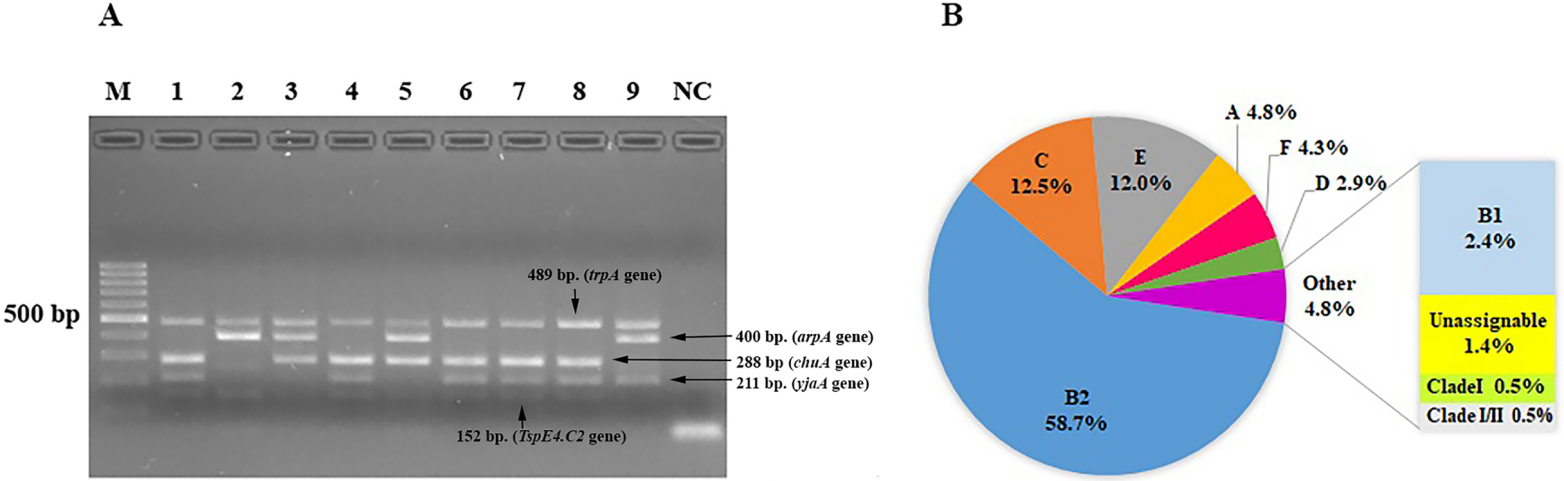
The distribution of phylogenetic groups among uropathogenic *Escherichia coli* isolates by the new Clermont phylo-typing method. (A) Multiplex PCR profiles for specific uropathogenic *Escherichia coli* isolates by detecting the *arpA* (400 bp), *chuA* (288 bp), *yjaA* (211 bp), and *TspE4.C2* (152 bp) genes. Lane M, 100-base pair ladder (Fermantas); Lane 1, group B2 (*-, +, +,* +); Lane 2, group B1 (*+, -, -,* +); Lane 3, group D or E (*+, +, -,*-); Lane 4, group B2 (*-, +, +,* +); Lane 5, group D or E (*+, +, -,*-); Lane 6, group B2 (*-, +, +,* +); Lane 7, group B2 (*-, +, +,* +); Lane 8, group B2 (*-, +, +,* +); Lane 9, group A or C (*+, -, +,* -); Lane NC, negative control. The *trpA* (489 bp) internal control gene appeared in all samples except the negative control. Distilled water without any DNA as negative controls was used in PCR experiments. (B) The percentage of phylogenetic groups among uropathogenic *Escherichia coli* isolates.

We characterized the phylogenetic groups of *E. coli* from urine specimens by detecting the *arpA* (400 bp), *chuA* (288 bp)*, yjaA* (211 bp)*,* and *TspE4.C2* (152 bp) genes using multiplex PCR (Fig. 1A). Primers specific for the *trpA* (489 bp) gene were added to all PCR reactions to provide an internal control. Groups C and E were classified by amplification of the *trpA* (219 bp) and *arpA* (301 bp) genes using specific primers. The majority of the 208 *E. coli* isolates were group B2 (122, 58.7%), followed by group C (26, 12.5%), group E (25, 12%), group A (10, 4.8%), group F (9, 4.3%), group D (6, 2.9%), group B1 (5, 2.4%), unassignable (3, 1.4%), and clade I or clade II (2, 1.0%; Fig. 1B).

Adhesin-encoding genes were successfully amplified by multiplex PCR. The most frequent UPEC adhesin gene was *fimH* (191, 91.8%), followed by *pap* (165, 79.3%), *sfa* (25, 12.0%), and *afa* (16, 7.7%). We also investigated the adhesin gene patterns of the strains (Table 2). Among the isolates, 30 (14.4%), 167 (80.3%), and 11 (5.3%) possessed 1, 2, and 3 adhesin genes, respectively. A high prevalence of combined *fimH* and *pap* genes was significantly found (69.2%, *p* <  0.0001). Moreover, the *fimH* gene has significant association with UPEC phylogenetic groups B2 (*p* = 0.041). There were significant associations between phylogenetic group E and two adhesin genes namely *pap* and *afa* (*p* = 0.002 and *p* <  0.0001, respectively). Similarly, there were significant associations between phylogenetic group F and adhesin genes *fimH* and *sfa* (*p* = 0.005 and *p* = 0.044, respectively) (See Table 3).

**Table 2:** Profiles of adhesin genes in uropathogenic *Escherichia coli* strains.

**No. of genes**	**Adhesin genes patterns**	**No. of isolates (%)**	**Chi-square**	**Degree of freedom**	***P*-value**
1 gene, *n* = 30 (14.4%)					
	*fimH*	19 (9.1)			
	*pap*	4 (1.9)			
	*sfa*	5 (2.4)			
	*afa*	2 (1.0)			
2 genes, *n* = 167 (80.3%)					
	*fimH, pap*	144 (69.2)	922.88^a^	10	<0.0001
	*fimH, sfa*	11 (5.3)			
	*fimH, afa*	6 (2.9)			
	*pap, sfa*	2 (1.0)			
	*pap, afa*	4 (1.9)			
3 genes, *n* = 11 (5.3%)					
	*fimH, pap, sfa*	7 (3.4)			
	*fimH, pap, afa*	4 (1.9)			

**Notes.**

a 0 cells (.0%) have expected frequencies less than 5. The minimum expected cell frequency is 18.9.

**Table 3:** The association between phylogenetic groups and adhesin genes of uropathogenic *Escherichia coli* isolates.

**Adhesin genes**		**Phylogenetic group**																			****
		B2 (*n* = 122)				C (*n* = 26)				E (*n* = 25)				A (*n* = 10)				F (*n* = 9)			
		B2	Non-B2	*χ*^**2**^	*P*-value	C	Non-C	*χ*^**2**^	*P*-value	E	Non-E	*χ*2	*P*-value	A	Non-A	*χ*^**2**^	*P*-value	F	Non-F	*χ*2	*P*-value
*fimH*	Present	**116**	**75**	**4.166**	**0.041**	24	167	0.009	0.924	22	169	0.554	0.456	10	181	0.935	0.334	**6**	**185**	**7.935**	**0.005**
	Absent	**6**	**11**			2	15			3	14			0	17			**3**	**14**		
*pap*	Present	100	65	1.254	0.263	20	145	0.105	0.746	**14**	**151**	**9.428**	**0.002**	10	155	2.738	0.098	7	158	0.014	0.907
	Absent	22	21			6	37			**11**	**32**			0	43			2	41		
*sfa*	Present	19	6	3.526	0.060	1	24	1.877	0.171	1	24	1.728	0.189	0	25	1.435	0.231	**3**	**22**	**4.041**	**0.044**
	Absent	103	80			25	158			24	159			10	173			**6**	**177**		
*afa*	Present	6	10	3.198	0.074	0	16	2.476	0.116	**8**	**8**	**23.645**	**0.000**	0	16	0.875	0.349	1	15	0.155	0.694
	Absent	116	76			26	166			**17**	**175**	****	****	10	182			8	184		
**Adhesin genes**		**Phylogenetic group**																			****
		**D (n=6)**				**B1 (n=5)**				**Unassignable (n=3)**				**CladeI (n=1)**				**Clade I or II (n=1)**			
		**D**	**Non-D**	***χ* 2**	***P*-value**	**B1**	**Non-B1**	***χ* 2**	***P*-value**	**Unassign**	**Non-unassign**	***χ* 2**	***P*-value**	**CladeI**	**Non-cladeI**	***χ* 2**	***P*-value**	**CladeI/II**	**Non-cladeI/II**	***χ* 2**	***P*-value**
*fimH*	Present	5	186	0.594	0.441	5	186	0.456	0.500	2	189	2.567	0.109	1	190	0.089	0.765	**0**	**191**	**11.290**	**0.001**
	Absent	1	16			0	17			1	16			0	17			**1**	**16**		
*pap*	Present	5	160	0.060	0.806	4	161	0.001	0.970	3	162	0.739	0.373	1	164	0.262	0.609	1	164	0.262	0.609
	Absent	1	42			1	42			0	43			0	43			0	43		
*sfa*	Present	0	25	0.844	0.358	1	24	0.309	0.579	0	25	0.416	0.519	0	25	0.137	0.711	0	25	0.137	0.711
	Absent	6	177			4	179			3	180			1	182			1	182		
*afa*	Present	0	16	0.515	0.473	0	16	0.427	0.513	1	15	2.818	0.093	0	16	0.084	0.772	0	16	0.084	0.772
	Absent	6	186			5	187			2	190			1	191			1	191		

We performed antimicrobial susceptibility tests on *E. coli* strains using different categories of antibiotics. There were significant associations between *E. coli* phylogenetic groups and resistance rates of antibiotics (*p* <  0.05) except ampicillin, gentamicin and trimethoprim-sulfamethoxazole (Table 4). All isolates showed high rates of resistance to ampicillin (84.1%), ciprofloxacin (65.4%), norfloxacin (65.4%), levofloxacin (64.9%), trimethoprim-sulfamethoxazole (54.3%), cefazolin (44.7%), cefotaxime (43.8%), ceftriaxone (43.8%), ceftazidime (43.8%), amoxicillin-clavulanate (36.5%), and gentamicin (33.7%). The rates of resistance to other antibiotics were between ∼1% and 6%. *E. coli* phylogenetic group C had the highest rates of resistance to all antibiotics (*p* <0.05) except ampicillin, gentamicin, amikacin, netilmicin, and fosfomycin (Table S3). Three isolates (1.4%) in group C were carbapenems-resistant. Interestingly, most of the 129 isolates (62.0%) that were MDR and belonged to group B2 (59.7%; 77 of 129). However, the lower resistance rates to piperacillin-tazobactam and carbapenems were observed in group B2 (*p* = 0.005 and *p* = 0.0038, respectively) (Table S3). The lowest rates of resistance to cephalosporin were observed in group A (*p* = 0.02), while group D was more susceptible to fluoroquinolones than the other groups (*p* = 0.01). The only one isolate of group A was resistant to fosfomycin (*p* <  0.0001).

**Figure 2:**
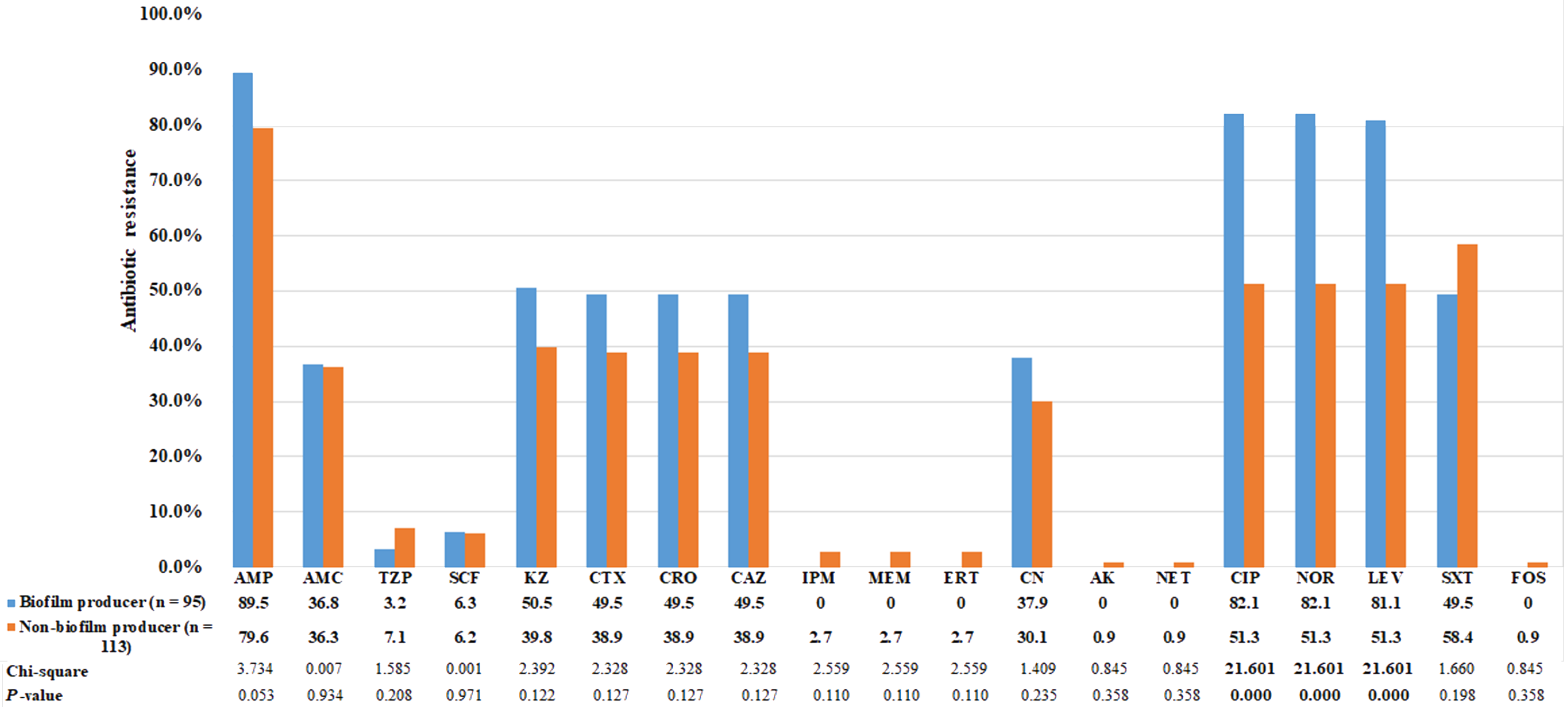
Comparison of antibiotic resistance between biofilm producers and non-biofilm producers. Uropathogenic *E. coli* strains were evaluated for in vitro susceptibility to nineteen antibiotics: Amp, ampicillin; AMC, amoxicillin-clavulanic acid; TZP, piperacillin-tazobactam; SCF, cefoperazone-sulbactam; KZ, cefazolin; CTX, cefotaxime; CRO, ceftriaxone; CAZ, ceftazidime; CN, gentamicin; CIP, ciprofloxacin; NOR, norfloxacin; LEV, levofloxacin; SXT, trimethoprim-sulfamethoxazole; IPM, Imipenem; MEM, meropenem; ERT, ertapenem; CN, gentamicin; AK, amikacin; NET, netilmicin; FOS, fosfomycin. Bar graphs show the percentage of antibiotic resistance among biofilm producers in blue and non-biofilm producers in orange.

Using the CRA method, the abilities of bacteria to form biofilm were categorized into four groups based on the color tones of colonies. Among the 95 *E. coli* strains that could form biofilm, 4 (4.2%) showed strong biofilm-forming ability, 38 (40.0%) showed moderate ability, and 53 (55.8%) showed weak ability. The biofilm-producing strains were predominantly clustered in phylogenetic group B2 (Table 5). Biofilm- and non-biofilm-producing UPEC showed different antimicrobial resistance profiles. Among the biofilm producers, the rate of resistance was highest for ampicillin (90%), followed by fluoroquinolones (82%), cephalosporins (50%), and gentamicin (38%). No biofilm producer was resistant to carbapenems. In contrast, the non-biofilm producers were more resistant to TMP-SMZ (58%), followed by piperacillin-tazobactam (7%) and carbapenems (3%). The frequency distribution is presented in Fig. 2. The resistance rates to ciprofloxacin, norfloxacin and levofloxacin among biofilm producers were significantly higher than non-biofilm producers (*p* <  0.0001; Fig. 2). Of the 129 MDR *E. coli* isolates, 54% were biofilm producers.

**Table 4:** Chi-square test for comparisons of resistance rates to antimicrobial agents among various phylogenetic groups of uropathogenic *Escherichia coli* isolates.

**Antimicrobial resistance rates**	**Phylogenetic group**										**Chi- square**	***P*-value**
	B2	C	E	A	F	D	B1	Unassignable	Clade I and I or II	Total		
	*n* = 122(%)	*n* = 26(%)	*n* = 25(%)	*n* = 10(%)	*n* = 9(%)	*n* = 6(%)	*n* = 5(%)	*n* = 3(%)	*n* = 2(%)	*n* = 208(%)		
**Penicillins**												
AMP	101 (82.8)	25 (96.2)	21 (84)	8 (80)	6 (66.7)	6 (100)	4 (80)	3 (100)	1 (50)	175 (84.1)	16.707	0.054
***β*-lactam/ *β*-lactamase inhibitor combinations**												
AMC	39 (32)	17 (65.4)	9 (36)	3 (30)	4 (44.4)	1 (16.7)	3 (60)	0	0	76 (36.5)	16.906	**0.050**
TZP	2 (1.6)	7 (26.9)	1 (4)	0	1 (11.1)	0	0	0	0	11 (5.3)	29.961	**0.000**
SCF	5 (4.1)	7 (26.9)	1 (4)	0	0	0	0	0	0	13 (6.3)	22.477	**0.007**
**Cephalosporins**												
KZ	54 (44.3)	18 (69.2)	11 (44)	1 (10)	5 (55.6)	2 (33.3)	1 (20)	1 (33.3)	0	93 (44.7)	15.248	**0.084**
CTX	53 (43.4)	18 (69.2)	11 (44)	1 (10)	5 (55.6)	1 (16.7)	1 (20)	1 (33.3)	0	91 (43.8)	16.977	**0.049**
CRO	53 (43.4)	18 (69.2)	11 (44)	1 (10)	5 (55.6)	1 (16.7)	1 (20)	1 (33.3)	0	91 (43.8)	16.977	**0.049**
CAZ	52 (42.6)	18 (69.2)	11 (44)	1 (10)	5 (55.6)	1 (16.7)	2 (40)	1 (33.3)	0	91 (43.8)	16.977	**0.049**
**Carbapenems**												
IPM	0	3 (11.5)	0	0	0	0	0	0	0	3 (1.4)	21.307	**0.011**
MEM	0	3 (11.5)	0	0	0	0	0	0	0	3 (1.4)	21.307	**0.011**
ERT	0	3 (11.5)	0	0	0	0	0	0	0	3 (1.4)	21.307	**0.011**
**Aminoglycosides**												
CN	43 (33.6)	12 (46.2)	9 (36)	1(10)	4 (44.4)	1 (16.7)	1 (20)	0	0	70 (33.7)	10.759	0.293
AK	0	0	0	0	1 (11.1)	0	0	0	0	1 (0.5)	25.121	**0.003**
NET	0	0	0	0	1 (11.1)	0	0	0	0	1 (0.5)	25.121	**0.003**
**Fluoroquinolones**												
CIP	87 (71.3)	25 (96.2)	10 (40)	4 (40)	5 (55.6)	1 (16.7)	2 (40)	2 (66.7)	0	136 (65.4)	36.148	**0.000**
NOR	88 (72.1)	25 (96.2)	10 (40)	4 (40)	5 (55.6)	1 (16.7)	1 (20)	2 (66.7)	0	136 (65.4)	36.148	**0.000**
LEV	86 (70.5)	25 (96.2)	10 (40)	4 (40)	5 (55.6)	1 (16.7)	2 (40)	2 (66.7)	0	135 (64.9)	35.411	**0.000**
**Folate pathway inhibitors**												
SXT	61 (50)	20 (76.9)	16 (64)	5 (50)	5 (55.6)	4 (66.7)	0	1 (33.3)	0	113 (54.3)	10.853	0.286
**Fosfomycins**												
FOS	0	0	0	1 (10)	0	0	0	0	0	1 (0.5)	19.896	**0.019**

**Notes.**

Amp ampicillin AMC amoxicillin-clavulanic acid TZP piperacillin-tazobactam SCF cefoperazone-sulbactam KZ cefazolin CTX cefotaxime CRO ceftriaxone CAZ ceftazidime CN gentamicin CIP ciprofloxacin NOR norfloxacin LEV levofloxacin SXT trimethoprim-sulfamethoxazole IPM Imipenem MEM meropenem ERT ertapenem CN gentamicin AK amikacin NET netilmicin FOS fosfomycin

**Table 5:** Biofilm forming ability among various phylogenetic groups of uropathogenic *Escherichia coli* isolates.

**Phylogenetic group**	**Prevalence of biofilm formation ability**			
	**Strong (*n* = 4), %**	**Moderate (*n* = 38), %**	**Weak (*n* = 53), %**	**Absent (*n* = 113), %**
B2 (*n* = 122)	3 (2.5)	36 (29.5)	46 (37.7)	37 (30.3)
C (*n* = 26)	0	0	3 (11.5)	23 (88.5)
E (*n* = 25)	0	0	1 (4)	24 (96)
A (*n* = 10)	0	0	0	10 (100)
F (*n* = 9)	0	1 (11.1)	1 (11.1)	7 (77.8)
D (*n* = 6)	0	0	0	6 (100)
B1 (*n* = 5)	1 (20)	0	1 (20)	3 (60)
Unassignable (*n* = 3)	0	1 (33.3)	0	2 (66.7)
Clade I (*n* = 1)	0	0	0	1 (100)
Clade I or II (*n* = 1)	0	0	1 (100)	0

We also investigated the association between the presence or absence of the four adhesin genes and biofilm formation ability. The results demonstrated that biofilm production was significantly correlated with the presence of *pap* adhesin gene (*p* ≤ 0.05; Table 6). Among the biofilm producer group, we found the prevalence of *pap* gene was lower in strong biofilm formers than in weak and moderate.

**Table 6:** Prevalence of virulence genes among various groups of different biofilm formation ability.

**Virulence**	**Percentage of biofilm formation ability**								
**genes**	**Strong** **(*n* = 4), %**		**Moderate** **(*n* = 38), %**		**Weak** **(*n* = 53), %**	**Total** **(*n* = 95), %**	**Absent** **(*n* = 113), %**	**Pearson** **Chi-square**	***p*-value**
*fimH*	4 (100)		37 (97.4)		49 (92.5)	90 (94.7)	101 (89.4)	1.97^a^	0.16
*pap*	2 (50)		35 (92.1)		44 (83.0)	81 (85.3)	84 (74.3)	3.76^b^	**0.05**
*sfa*	0		1 (2.6)		6 (11.3)	7 (7.4)	18 (15.9)	3.58^c^	0.06
*afa*	0		2 (5.3)		3 (5.7)	5 (5.3)	11 (9.7)	1.45^d^	0.23

**Notes.**

*P*-values were calculated using the Pearson Chi-squared test. *P*-values ≤ 0.05 are indicated in bold.

a 0 cells (.0%) have expected count less than 5. The minimum expected count is 7.76.

b 0 cells (.0%) have expected count less than 5. The minimum expected count is 19.64.

c 0 cells (.0%) have expected count less than 5. The minimum expected count is 11.42.

d 0 cells (.0%) have expected count less than 5. The minimum expected count is 7.31.

## Discussion

The higher proportion of UTIs in female (74%) than male (26%) patients in this study was observed. This is most likely to the anatomical structure of the female urethra, which is shorter, wider, and closer to the anus than that of males. *E. coli* is common in the gastrointestinal tract flora and can be easily moved from the anus to the urinary tract, leading to UTIs (Dadi et al., 2020). Half of the UTI cases in this study (50%) were observed in female patients over 65 years of age. In postmenopausal women, the low level of estrogen and high intravaginal pH are associated with increased bacterial adherence to the uroepithelium cell, which causes UTIs (Johansson et al., 1996; Beyer et al., 2001). Our study included a large number of catheter urine specimens, which was correlated with the high percentage of infections in the over-65 age group. The low immunity level in the elderly puts those of advanced age at a high risk of bacterial infection and is responsible for the high prevalence in catheterized cases (Themphachanal et al., 2015).

Phylogenetic groups B2 and D are common strains implicated in UTIs (Ejrnæs et al., 2011). In contrast to the results of studies from Uruguay and Southern Thailand, where high prevalences of phylogenetic group D were found (Themphachanal et al., 2015; Robino et al., 2014), we observed that group B2 was the most prevalent UPEC (58.7%), followed by group C (12.5%). Our results are in accordance with several studies in which the dominant strain was found to be group B2. These studies were conducted in North America (45% prevalence of group B2) (Johnson et al., 2003), Denmark (67%) (Ejrnæs et al., 2011), Poland (35%) (Kot et al., 2016), South Korea (79%) (Lee et al., 2016), and Ethiopia (30%) (Dadi et al., 2020). Using a novel PCR-based method (Clermont et al., 2013), we could classify UPEC into groups C, E, and F and clade I, resulting in a lower percentage of strains in groups A, B1, and D than in earlier studies. This finding indicates that the triplex method of phylo-grouping misidentifies groups C, E, and F and clade I as belonging to group A, B1, B2, or D (Kumar, Nahid & Zahra, 2017). It had been reveal that some strains (1.4%) could not be assigned to a phylogenetic group due to simply relying upon PCR of a few small number of genes. As stated by Clermont et al. (2013), the unassignable strains are more likely the result of large-scale recombination events from two different groups or genome plasticity driven by loss and gain of genes. In this study, 1% of UPEC belonged to cryptic clade I/II. This is a much lower percentage than in a study conducted in Mexico (9%) (Kumar, Nahid & Zahra, 2017). The cryptic clades are primarily associated with environmental *E. coli*; thus, the observed results may be related to a lack of good hygiene practices. The different distributions of phylogenetic groups may depend on the geographic area, health status of the host, use of antibiotics, and/or variations in research design and sample size of the studies (Derakhshandeh et al., 2013).

The most prevalent adhesin gene was *fimH*, followed by *pap*, *sfa*, and *afa*. In agreement with studies conducted in Ethiopia (Dadi et al., 2020) and Iran (Tajbakhsh et al., 2016), phylogenetic group B2 strains showed the highest frequency of the adhesin genes in our study. We found a coexistence of *fimH* and *pap* genes (69.2%), indicating a high presence of virulence genes among UPEC isolated from UTI patients in Thailand. This outcome was different from that of a study in Iran, in which the combination of *pap* and *afa* virulence genes was more common (Rahdar et al., 2015). The ability of UPEC to form biofilm is a crucial virulence property. We found that 45.7% of UPEC were biofilm producers and that most of these classified into phylogenetic group B2. This finding demonstrates that biofilm formation may be associated with phylogenetic group B2. The association between biofilm-forming ability and some adhesin genes among UPEC was previously reported (Rahdar et al., 2015; Tajbakhsh et al., 2016; Naves et al., 2008). Consistently, the most significant correlation observed in our study was the correlation between the *pap* gene and biofilm production. The negative correlation found closely to significance between *sfa* gene and biofilm formation ( *p* = 0.06), as the prevalence of this gene was lower in biofilm producer. In contrast, no significant correlation was seen between the *fimH*, or *afa* genes and biofilm production in the strains evaluated in this study. This finding is in agreement with other studies that did not find significant correlations in clinical isolates of pathogenic *E. coli* (Reisner et al., 2006; Hancock, Ferrie‘res & Klemm, 2007). The discrepant results imply that these genes are not the only determinants of biofilm production in UPEC strains; rather, environmental and genetic factors may also be involved (Reisner et al., 2006). Adhesin genes such as *fimH* are under strict control by phase variation in many strains. The presence of adhesin genes certainly does not imply their expression. It would have been far more informative if the further study had been able to correlate expression of these genes rather than just their presence or absence by PCR.

It is important to perform antimicrobial susceptibility testing to select the appropriate empiric antibiotic therapy for UTIs. Our findings showed that the rate of resistance to ampicillin (84.1%) was higher than rates of resistance to other antibiotics. In general, fluoroquinolones are recommended for oral antimicrobial therapy in uncomplicated pyelonephritis. TMP-SMZ is commonly used in the treatment of uncomplicated cystitis, while AMC was a first-line therapy for complicated UTIs (Bonkat et al., 2019). However, our results revealed that rates of resistance to fluoroquinolones, TMP-SMZ, and AMC were 65%, 54%, and 37%, respectively. This result is consistent with a previous mini-review reporting increases in resistance rates of those drugs among UPEC isolates in developing countries (Kot, 2019). This likely emerged due to the widespread use of fluoroquinolones for uncomplicated UTIs or the inappropriate use of TMP-SMZ for empiric UTI treatment (Bartoletti et al., 2016). In this study, the strains in phylogenetic group C showed the highest rates of antibiotic resistance. In recent decades, the increasing rate of MDR in UPEC has become a public health threat. A high prevalence of MDR UPEC of approximately 62% was observed in the current study, similar to the findings reported in Iran (60.2%) (Tajbakhsh et al., 2016) and Nepal (63.2%) (Ganesh et al., 2019). The majority of MDR UPEC belonged to phylogenetic group B2, consistent with the outcomes reported in South Korea (73%) (Lee et al., 2016).

The present study found that biofilm producer strains were more resistant to ciprofloxacin, norfloxacin and levofloxacin than non-biofilm producers. These results were in agreement with previous studies indicating that the sessile bacterial cells are much less susceptible to antimicrobial agents than nonattached (planktonic) cells (Costerton, Stewart & Greenberg, 1999). A higher rate of resistance to TMP-SMZ was found among the non-biofilm producers than among the biofilm producers. One explanation for this finding is that these strains may carry the *dhfr* or *dhps* gene mutation on chromosomal DNA, which are common causes of resistance to this drug (Huovinen et al., 1995).

In conclusion, the majority of UPEC among patients with UTIs in this geographical area belonged to phylogenetic group B2. UPEC in this group also showed the highest prevalence of adhesin genes and biofilm formation. The analysis of the antimicrobial resistance of strains tested in this study showed a high level of resistance to cephalosporins, fluoroquinolones, TMP-SMZ, and AMC among strains belonging to groups B2 and C. Therefore, further study of the molecular epidemiology of UPEC and their antibiotic susceptibility patterns will improve our understanding of the organism and lead to a better management of UTIs.

##  Supplemental Information

10.7717/peerj.10453/supp-1 Supplemental Information 1 Sequence of oligonucleotide primers used for detection of the phylogenetic groups

10.7717/peerj.10453/supp-2 Supplemental Information 2 Sequence of oligonucleotide primers used for amplification of the adhesin genes

10.7717/peerj.10453/supp-3 Supplemental Information 3 The association between phylogenetic group and antimicrobial susceptibility patterns of uropathogenic *Escherichia coli* isolates

10.7717/peerj.10453/supp-4 Supplemental Information 4 Multiplex PCR profiles for specific uropathogenic *Escherichia coli* isolates according to the new Clermont phylo-typing method (Uncropped)

10.7717/peerj.10453/supp-5 Supplemental Information 5 The biofilm ability of uropathogenic Escherichia coli were determined by Congo Red Agar R, red; AB, almost black; VB, very black. The color tones of colonies were categorized as follows: very black, and almost black, which were interpreted as strong, and weak biofilm producers, respectively, and red reported as non-biofilm producers.

10.7717/peerj.10453/supp-6 Supplemental Information 6 Patient data and raw data for statistical analysis

## Additional Information and Declarations

### Data Availability

The following information was supplied regarding data availability: Raw data are available in the Supplementary Files.
