# Supplementary material for: Distribution of phylogenetic groups, adhesin genes, biofilm formation, and antimicrobial resistance of uropathogenic Escherichia coli isolated from hospitalized patients in Thailand
Source: PeerJ. 2020 Dec 2;8:e10453. doi: 10.7717/peerj.10453 (PMC7718785; doi:10.7717/peerj.10453)
Supplement: Supplemental Information 1 [file peerj-08-10453-s001.docx]

**Table S1** Sequence of oligonucleotide primers used for detection of the phylogenetic groups (Clermont et al., 2013)

| **PCR reaction** | **Target gene** | **Primer ID** | **Primer sequences (5′-3′)** | **Product size (bp)** |
| --- | --- | --- | --- | --- |
| Quadruplex | *chuA* | chuA.1b | ATGGTACCGGACGAACCAAC | 288 |
|  |  | chuA.2 | TGCCGCCAGTACCAAAGACA |  |
|  | *yjaA* | yjaA.1b | CAAACGTGAAGTGTCAGGAG | 211 |
|  |  | yjaA.2b | AATGCGTTCCTCAACCTGTG |  |
|  | *TspE4.C2* | TspE4C2.1b | CACTATTCGTAAGGTCATCC | 152 |
|  |  | TspE4C2.2b | AGTTTATCGCTGCGGGTCGC |  |
|  | *arpA* | AceK.f | AACGCTATTCGCCAGCTTGC | 400 |
|  |  | ArpA1.r | TCTCCCCATACCGTACGCTA |  |
| Group E | *arpA* | ArpAgpE.f | GATTCCATCTTGTCAAAATATGCC | 301 |
|  |  | ArpAgpE.r | GAAAAGAAAAAGAATTCCCAAGAG |  |
| Group C | *trpA* | trpAgpC.1 | AGTTTTATGCCCAGTGCGAG | 219 |
|  |  | trpAgpC.2 | TCTGCGCCGGTCACGCCC |  |
| Internal control | *trpA* | trpBA.f | CGGCGATAAAGACATCTTCAC | 489 |
|  |  | trpBA.r | GCAACGCGGCCTGGCGGAAG |  |

Clermont O, Christenson JK, Denamur E, Gordon DM. 2013. The Clermont *Escherichia coli* phylo-typing method revisited: improvement of specificity and detection of new phylo- groups. *Environmental microbiology reports* 5(1):58–65. DOI: 10.1111/1758-2229.12019
