# Supplementary material for: Distribution of phylogenetic groups, adhesin genes, biofilm formation, and antimicrobial resistance of uropathogenic Escherichia coli isolated from hospitalized patients in Thailand
Source: PeerJ. 2020 Dec 2;8:e10453. doi: 10.7717/peerj.10453 (PMC7718785; doi:10.7717/peerj.10453)
Supplement: Supplemental Information 2 [file peerj-08-10453-s002.docx]

**Table S2** Sequence of oligonucleotide primers used for amplification of the adhesin genes

| Gene | Primer sequence (5′-3′) | Product size  (bp) | Reference |
| --- | --- | --- | --- |
| *pap* | GCAACAGCAACGCTGGTTGCATCAT | 336 | Yamamoto et al., 1995 |
|  | AGAGAGAGCCACTCTTATACGGACA |  |  |
| *sfa* | CTCCGGAGAACTGGGTGCATCTTAC | 410 | Le Bouguenec et al., 1992 |
|  | CGGAGGAGTAATTACAAACCTGGCA |  |  |
| *afa* | GCTGGGCAGCAAACTGATAACTCTC | 750 | Le Bouguenec et al., 1992 |
|  | CATCAAGCTGTTTGTTCGTCCGCCG |  |  |
| *fimH* | GAGAAGAGGTTTGATTTAACTTATTG | 559 | Struve & Krogfelt, 1999 |
|  | AGAGCCGCTGTAGAACTGAGG |  |  |

# References

Le Bouguenec C, Archambaud M, Labigne A. 1992. Rapid and specific detection of the *pap*, *afa*, and *sfa* adhesin-encoding operons in uropathogenic *Escherichia coli* strains by polymerase chain reaction. *Journal of clinical microbiology* 30(5):1189–1193.

Struve C, Krogfelt KA. 1999. In vivo detection of *Escherichia coli* type 1 fimbrial expression and phase variation during experimental urinary tract infection. *Microbiology* 145(Pt 10):2683–2690. DOI: 10.1099/00221287-145-10-2683.

Yamamoto S, Terai A, Yuri K, Kurazono H, Takeda Y, Yoshida O. 1995. Detection of urovirulence factors in *Escherichia coli* by multiplex polymerase chain reaction. *FEMS immunology and medical microbiology* 12(2):85–90.

DOI: 10.1111/j.1574-695X.1995.tb00179.x.
