# Supplementary material for: Distribution of phylogenetic groups, adhesin genes, biofilm formation, and antimicrobial resistance of uropathogenic Escherichia coli isolated from hospitalized patients in Thailand
Source: PeerJ. 2020 Dec 2;8:e10453. doi: 10.7717/peerj.10453 (PMC7718785; doi:10.7717/peerj.10453)
Supplement: Supplemental Information 4 [file peerj-08-10453-s004.docx]

**Figure S1** Multiplex PCR profiles for specific uropathogenic *Escherichia coli* isolates according to the new Clermont phylo-typing method. (Uncrop)


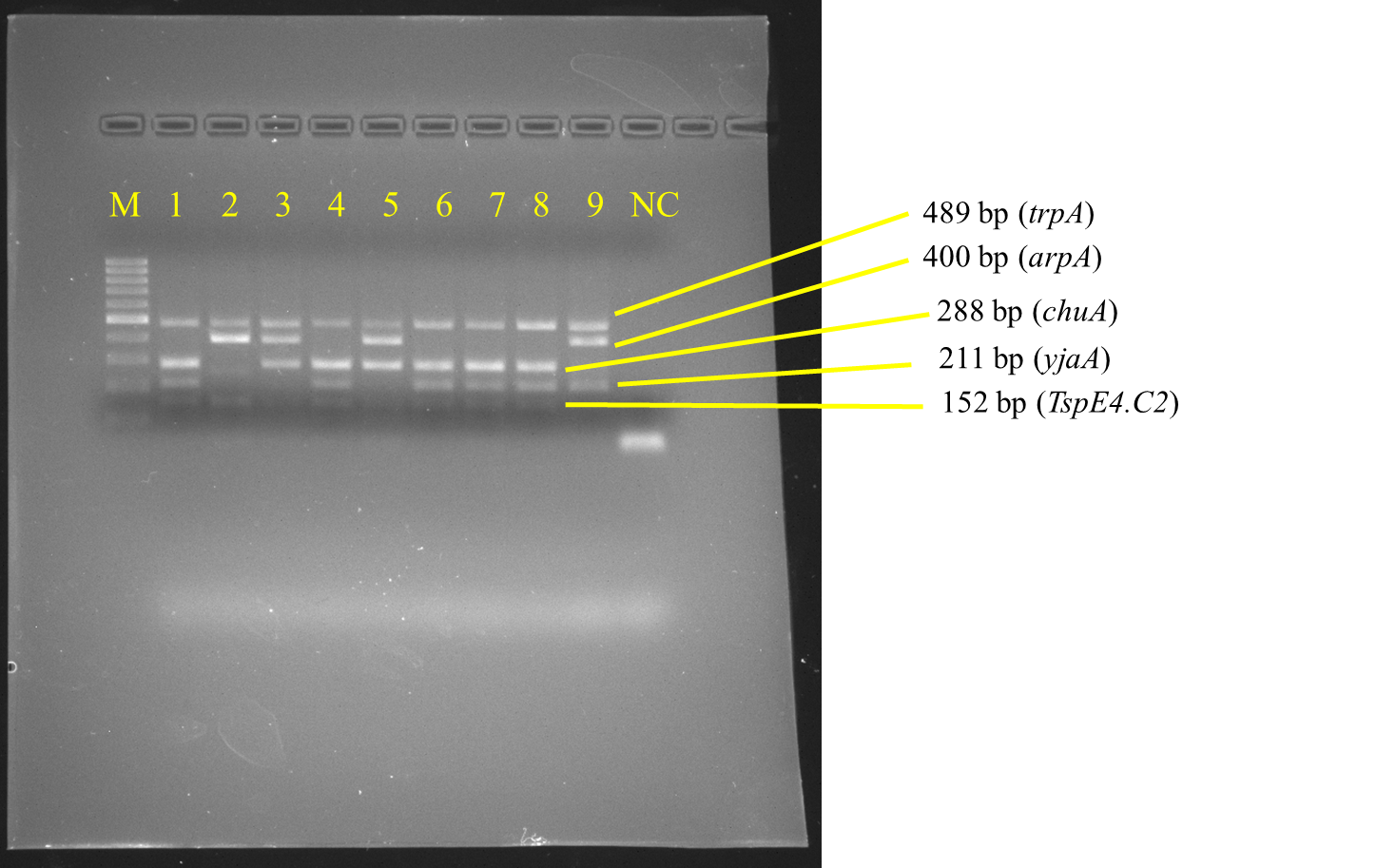


Multiplex PCR profiles for specific uropathogenic *Escherichia coli* isolates by detecting the *arpA* (400 bp), *chuA* (288 bp), *yjaA* (211 bp), and *TspE4.C2* (152 bp) genes. Lane M, 100-base pair ladder (Fermantas); Lane 1, group B2 (*-, +, +,* +); Lane 2, group B1 (*+, -, -,* +); Lane 3, group D or E (*+, +, -,* -); Lane 4, group B2 (*-, +, +,* +); Lane 5, group D or E (*+, +, -,* -); Lane 6, group B2 (*-, +, +,* +); Lane 7, group B2 (*-, +, +,* +); Lane 8, group B2 (*-, +, +,* +); Lane 9, group A or C (*+, -, +,* -); Lane NC, negative control. The *trpA* (489 bp) internal control gene appeared in all samples except the negative control.
