# Supplementary material for: Distribution of phylogenetic groups, adhesin genes, biofilm formation, and antimicrobial resistance of uropathogenic Escherichia coli isolated from hospitalized patients in Thailand
Source: PeerJ. 2020 Dec 2;8:e10453. doi: 10.7717/peerj.10453 (PMC7718785; doi:10.7717/peerj.10453)
Supplement: Supplemental Information 5 — R, red; AB, almost black; VB, very black. The color tones of colonies were categorized as follows: very black, and almost black, which were interpreted as strong, and weak biofilm producers, respectively, and red reported as non-biofilm producers. [file peerj-08-10453-s005.docx]

**Figure S2** Detection the biofilm ability of uropathogenic *Escherichia coli* by Congo Red Agar.


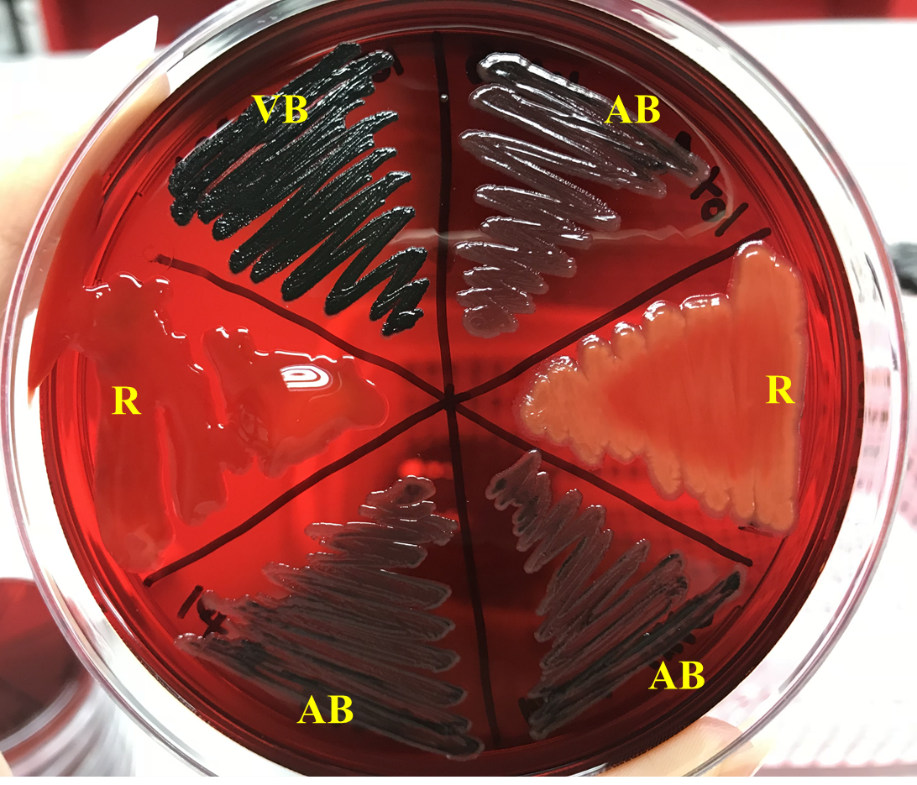


R, red; AB, almost black; VB, very black. The color tones of colonies were categorized as follows: very black, and almost black, which were interpreted as strong, and weak biofilm producers, respectively, and red reported as non-biofilm producers.
